# Prevalence and patterns of gender-based violence in Enugu, Nigeria: a cross-sectional study

**DOI:** 10.11604/pamj.2022.41.198.29454

**Published:** 2022-03-11

**Authors:** Onyinye Hope Chime, Obinna Chukwuebuka Nduagubam, Chinonyelu Jennie Orji

**Affiliations:** 1 Department of Community Medicine, College of Medicine, Enugu State University of Technology, Enugu, Nigeria,; 2 Department of Paediatrics, College of Medicine, Enugu State University of Technology, Enugu, Nigeria

**Keywords:** Gender-based violence, pandemic, Enugu

## Abstract

**Introduction:**

gender-based violence remains one of the most persistent human rights abuse in the world and with the emergence of the COVID-19 pandemic and its attendant mitigating protocols; Gender-based violence (GBV) could be on the rise with changes in its pattern and presentation. The aim of the study was to determine the prevalence and patterns of GBV among victims presenting in a tertiary health facility in South-East Nigeria during the first phase of the COVID-19 pandemic.

**Methods:**

this was a retrospective cross-sectional study, among 710 victims of GBV, who reported and received care at the GBV unit in a tertiary hospital in Enugu, South-east Nigeria. A pro forma designed by the researchers was used to collect secondary data from GBV facility screening forms and folders of all patients that reported any form of GBV over a 3-year period (2018 - 2020).

**Results:**

majority (89.9%) of victims of GBV were females and over a half (51.4%) of the cases were in the age group 20-39 years. In total, 56.8% of the GBV cases had been sexually abused. The pattern of GBV over the three years period under study revealed an increase in proportion for both sexual and physical/emotional violence, with a peak in 2019 and a reduction of cases in 2020. Persons below 19 years of age were 23 times more likely to experience sexual violence, than those between 40-59 years of age (AOR: 23.332; 95% CI: 11.037 -49.325) p<0.001. Males were 11 times more likely to experience physical/emotional violence than females (AOR: 11.136; 95% CI: 4.685-26.471) p<0.001. Age, gender, urban dwelling and year of occurrence were significant predictors of GBV.

**Conclusion:**

GBV is a cause for concern in Enugu Nigeria; affecting mainly young female victims in their prime. There is an increase in reported cases of GBV in Enugu Nigeria with sexual abuse being more prevalent.

## Introduction

According to the United Nations, “Gender-based violence (GBV) is an act of violence that results in physical, sexual, or psychological harm or suffering to women, girls, men, and boys, as well as threats of such acts, coercion, or the arbitrary deprivation of liberty whether occurring in public or in private life” [1-3]. GBV is a form of violence against an individual based on that person´s biological sex, gender identity or expression, or perceived adherence to socially-defined expectations of one´s gender [4]. It is the most persistent yet least evident human rights violation in the world over, cutting across age, gender, religion, social and economic boundaries [5]. GBV is characterized by the use and abuse of physical, emotional or financial power and control over victims, thereby deteriorating their health, dignity, confidence and sovereignty [4, 5].

This violence, rooted in gender-related power differences, includes but not limited to intimate partner violence, rape, sexual assault and sexual violence [4]. These various forms of GBV may not occur in isolation; rather, mutually. Survivors of GBV suffer devastating short and long-term effects on their physical and psychological health including various degrees of physical injuries, forced and unintended pregnancies, unsafe abortions, sexually transmitted infections including HIV, depression, anxiety, post-traumatic stress disorder, limited ability to complete daily tasks, and in severe cases, death [6]. Asides from health consequences, GBV has serious consequences on employment, productivity and overall economic development. Even with these consequences, GBV remains masked in a culture of silence due to perceived stigmatization from kin and friends. Many survivors in their bid to seek justice are blamed, face retribution or ostracized from their families and communities, thereby plummeting them further into poverty, isolation and additional violence [2, 5]. This discourages other survivors from disclosing GBV or seeking medical care, as culture places priority over protecting family honour and image over seeking justice for this heinous crime [7].

Acts of GBV cut across both genders. However, most cases of GBV involve a female survivor and a male perpetrator and even in cases of GBV against boys or men, the violence is also committed by male perpetrators [4-6]. Women and girls being vulnerable are at greater risk and are generally less able to avoid or escape abuse [4-6]. This has resulted in the term ‘GBV´ being used interchangeably with ‘violence against women´ although the former term has a broader meaning than the latter. World over, females have less control over their bodies, decisions and resources than males [5]. Approximately one in three women and girls worldwide will experience physical or sexual violence in their lifetime, mostly by an intimate partner [2, 3, 5]. Though this occurs among women around the globe, there are variations within and between countries, regions, communities and social classes [2]. GBV against women is high in some cultural settings with male preference, where women are perceived as the property of their husbands and considered second class citizens [8, 9]. The highest prevalence of physical or sexual intimate partner violence was reported in the African region, predominantly in sub-Saharan Africa (65.64%) [10]. According to the 2018 National Demographic Health Survey (NDHS), 33% of women age 15-49 in Nigeria have experienced physical or sexual violence; 24% have experienced only physical violence, 2% have experienced only sexual violence, while 7% have experienced both physical and sexual violence [1]. Aside from physical and sexual violence, other forms of violence reported by Nigerian women include; socio-economic and psychological violence. Others are harmful traditional practices and violence against civilian women in combats [8]. Several studies have reported that the prevalence of GBV is higher among females with low social-economic status, illiterate and countries with the weak legal system [5, 6, 11, 12]. In addition, in cultural settings where men use violence to restraint and control their women, gender inequality and violence are reinforced and perpetrated continually [5, 11].

Although GBV has been increasingly recognized as a public health problem, yet it has been largely ignored. Globally, as many as 38 per cent of victims (women) died at the hands of an intimate partners [7]. A two-year retrospective study (2014-2016) among survivors of GBV in Enugu reveals a 50.8% increase among girls aged 11-22 years, while a prevalence as high as 74.4% was recorded in survivors below 18 years in another study [9, 10]. Following a chain of reports on both physical and sexual violence against women during the COVID-19 pandemic lockdown, a state of emergency on GBV was declared in Nigeria in June 2020 resulting in the initiation of sex offender registries and stiffer punishment for offenders [13]. In view of the above, the study explored the prevalence and pattern of different forms of GBV recorded in Enugu metropolis from 2018 to 2020. Findings will provide information on the pattern of GBV in the state, reduce risk of GBV, enable survivors to access specialized care and support and also aid their recovery in the community.

## Methods

**Study design and population:** this was a retrospective cross-sectional study among survivors of GBV who received care at the GBV unit domiciled at the antiretroviral therapy (ART) clinic of Enugu State University Teaching Hospital (ESUTH), Enugu, South-east Nigeria.

**Study setting:** essential GBV care services are provided in this facility in line with the first line support defined by WHO as basic empathetic counselling using the acronym “LIVES” (Listening, Inquiring, Validating, Ensuring safety and Support), HIV and sexually transmitted infection (STI) screening, ART post-exposure prophylaxis for sexually assaulted survivors, emergency contraception and referrals to appropriate authorities such as the police or a non-governmental organization - TAMAR Sexual Assault Referral Centre (TAMAR-SAC) for further support services [4]. These GBV services, provided in a private space to ensure utmost confidentiality and privacy are free and readily available daily for clients within Enugu metropolis and its environs. The services are supported by ART implementing partners - the Catholic Caritas Foundation of Nigeria, with funds provided by the Presidential Emergency Plan for AIDS Relief (PERPFAR) in collaboration with the Federal Ministry of Health. Patients are occasionally referred from other units within the facility, from other health facilities, police, and non-governmental agencies.

**Data collection:** data collection was done over a period of three weeks between February and March 2021 by the researchers. Secondary data over a 3-year period (2018-2020) were collected from the Gender Based Violence facility based screening forms (in patients folders) and the Facility Post Gender Based Violence care register. Being a total population study, all data available were collected using a pro forma designed by the researchers, who were trained on how to collect the data. Records with incomplete data were excluded. Information elicited include the age of survivors, sex and area of residence. Other variables assessed were in relation to the violence; the month of abuse, year of abuse and type of GBV (sexual, physical and emotional). Sexual violence includes actual, attempted or threatened (vaginal, anal or oral) rape, including marital rape; sexual abuse and exploitation; forced prostitution; transactional/survival sex; and sexual harassment; physical violence includes actual, attempted or threatened physical assault or battery; and emotional violence includes abuse and humiliation, such as insults; cruel and degrading treatment [14].

**Data analysis:** data obtained were analysed using the Statistical Package for Social Sciences (SPSS version 23). Data were presented as frequencies and proportions. Bivariate analysis was used to determine factors associated with GBV in this study. Variables that had p-value less than 0.2 in the bivariate analysis were entered into the logistic regression model to determine the predictors of sexual violence and physical/emotional violence among the survivors. Results were reported using odds ratio, confidence interval at 95% and level of significance was set at p-value less than 0.05.

**Ethical consideration:** ethical approval for the study was obtained from the Health Research Ethics Committee of the Enugu State University Teaching Hospital (protocol number: ESUTHP/C-MAC/RA/034/VOL.2/29). Permission to collect data was sought and obtained from the ART project coordinator.

## Results

**Socio-demographic characteristics of the respondents:** over a half (51.4%) of the total number of reported cases of GBV occurred among the survivors in the age group 20-39 years. This was followed by 38.5% among survivors aged 1-19 years, who are mainly children. Most (89.9%) of the victims of GBV were females and more cases were from urban areas compared to rural. Higher numbers (47.9%) of cases of GBV were reported in the third quarter (July to September) of the year with the first quarter (January to March) having the least number of cases. More cases (53.1%) of GBV were reported in the year 2019 when compared to years 2020 and 2018. More cases of sexual based gender abuse (56.8%) were recorded compared to physical and emotion abuse (Table 1).

**Table 1:** socio-demographic characteristics of GBV survivors

Variable	Frequency (n=710)	Percentage (%)
**Age (years)**		
Mean Age ± SD	22.2 ± 11.5	
**Age categorized**		
1-19	273	38.5
20-39	365	51.4
40-59	72	10.1
**Sex**		
Male	72	10.1
Female	638	89.9
**Area**		
Urban	443	62.4
Rural	267	37.6
**Reported month of abuse**		
Jan-March	116	16.3
April-June	129	18.2
July-September	340	47.9
October- December	125	17.6
**Reported year of abuse**		
2018	120	16.9
2019	377	53.1
2020	213	30.0
**Gender-based Violence**		
Sexual based	403	56.8
Physical/Emotional	307	43.2

**Figure 1:**
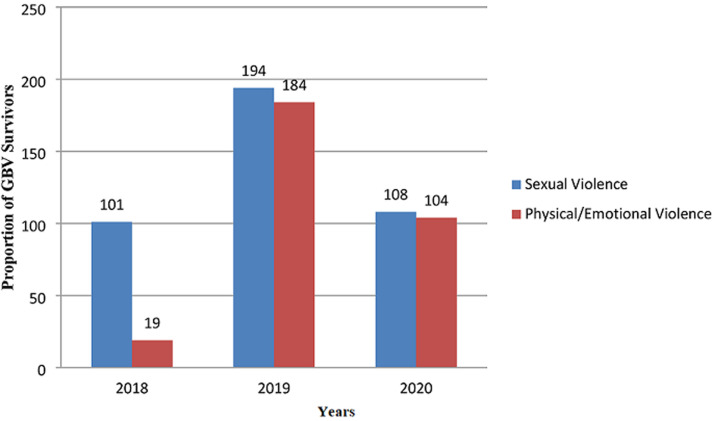
prevalence of gender based violence reported from 2018 to 2020

**Trend analysis of Gender based violence from 2018 to 2020:** the pattern of GBV over the three years period under study revealed an increasing frequency in both sexual and physical/emotional violence, with a peak in 2019 and a reduction of cases in 2020. However, the proportion of cases of physical/emotional violence (96.29%) to the total cases of GBV per year was higher in 2020 compared to 2019 and 2018 (Figure 1).

**Association between predictor factors and sexual violence:** young people less than 19 years of age were found to be 23 times more likely to experience sexual violence, than those between 40-59 years of age [Adjusted odds ratio (AOR): 23.332;95% Confidence interval (CI): 11.037 -49.325], p<0.001. Male survivors were about 9.4 times less likely to experience sexual violence than the females. (AOR: 0.106; 95%CI: 0.046-0.244), p<0.001. The year 2020 witnessed a 3.6 times greater surge in sexual violence than other years under study (AOR: 3.614; 95% CI: 1.770-7.381), p<0.001 (Table 2).

**Table 2:** predictors of sexual violence among the GBV survivors

Variable	Yes (n=403)	No (n=307)	P-value from multivariate analysis*	AOR {95% CI]**
**Age (years)**				
1-19	239 (59.3)	34 (11.1)	<0.001	23.332 (11.037-49.325)
20-39	152 (37.7)	213 ( (69.4)	0.019	2.280 (1.146-4.537)
40-59	12 (3.0)	60 (19.5)		1
**Gender**				
Male	10 (2.5)	62 (20.2)	<0.001	0.106 (0.046-0.244)
Female	393 (97.5)	245 (79.8)		1
**Area**				
Urban	271 (67.2)	172 (56.0)	0.058	1.447 ((0.988-2.118)
Rural	132 (32.8)	135 (44.0)		1
**Reported month of abuse**				
Jan-March	64 (15.9)	52 (16.9)	0.667	1.163 (0.586-2.308)
April-June	72 (17.9)	57 (18.6)	0.632	1.178 (0.602-2.307)
July-September	185 (45.9)	155 (50.5)	0.469	0.804 (0.602-2.307)
October- December	82 (20.3)	4 3 (14.0)		1
**Reported year of abuse**				
2020	108 (26.8)	105 (34.2)	<0.001	3.614 (1.779-7.381)
2019	194 (48.1)	183 (59.6)	0.439	0.836 (0.532-1.315)
2018	101 (25.1)	19 (6.2)		1

p-value on multivariate,* AOR-Adjusted Odds Ratio at 95% Confidence Interval,** p-value <0.2 at bivariate were logged into multiple logistic regression model

**Association between predictor factors and physical/emotional violence:** while males were 11 times more likely to experience physical/emotional violence than females (AOR: 11.136; 95% CI: 4.685-26.471) p<0.001, the year 2019 experienced a 1.2times higher reporting of physical/emotional violence than the years 2018 and 2020. (AOR: 1.22; 95% CI: 0.777-1.926) p<0.001 (Table 3).

**Table 3:** predictors of physical/emotional violence among the GBV survivors

Variable	Yes (n=307)	No (n=403)	P-value from multivariate analysis*	AOR {95% CI]**
**Age (years)**				
1-19	34(11.1)	239 (59.3)	<0.001	0.038 (0.018-0.082)
20-39	212 (69.1)	153 (38.0)	0.009	0.391 (0.193-0.794)
40-59	61 (19.9)	11 (2.7)		1
**Gender**				
Male	63 (20.5)	9 (2.2)	<0.001	11.136 (4.685-26.471)
Female	244 (79.5)	394 (97.8)		1
**Area**				
Urban	173 (56.4)	270 (67.0)	0.090	0.719 (0.490-1.053)
Rural	134 (43.6)	133 (33.0)		1
**Reported month of abuse**				
Jan-March	55 (17.9)	61 (15.1)	0.974	1.012 (0.508-2.016)
April-June	56 (18.2)	73 (18.1)	0.557	0.817(0.416-1.604)
July-September	153 (49.8)	187 (46.4)	0.504	1.225 (0.676-2.220)
October- December	43 (14.0)	82 (20.3)		1
**Reported year of abuse**				
2018	19 (6.2)	101 (25.1)	0.001	0.288 (0.141-0.590)
2019	184 (54.9)	193 (47.9)	0.385	1.223 (0.777-1.926)
2020	104 (33.9)	109 (27.0)		1

p-value on multivariate,* AOR-Adjusted Odds Ratio at 95% Confidence Interval,** p-value <0.2 at bivariate were logged into multiple logistic regression model

## Discussion

Over a half of the total number of reported cases of GBV occurred among survivors in the age group 20-39 years, followed closely by cases reported among persons aged below 19 years who were mainly children. These are people in their prime and earlier studies have reported similar higher incidence of GBV in people of similar age bracket [15-17]. Most of the victims of GBV in this study were females. Although both genders experience violence, studies have reported that females are more prone to GBV compared to males [18-21]. Fraiyal *et al*. in their study ascribed this to the age long patriarchal philosophy that men have more power and privilege than women [22].

Globally, about 1 in 3 women experience one form of GBV in their lifetime and that sexual violence was reported more than physical and emotional violence [23, 24]. In this study, sexual violence was also reported more than physical and emotional violence. In addition, Fawole *et al*. reported that sexual violence was the most commonly reported form of GBV compared to physical and psychological violence in Abuja, Nigeria [24].

In this study, more cases of GBV were from urban areas compared to rural. This may not be unconnected with the level of exposure of persons in the urban areas and their willingness to present to the facility and offer information about acts of violence against them compared to the rural, even when they in the rural areas may be more affected by GBV. Even if they are willing to report these crimes, they might not have the same level of information about the availability of such services. These services are mostly available in urban settings, which make access difficult for those in the rural settings and could one of the reasons why victims of GBV in urban areas are more likely to report their experiences than those from rural areas. This is supported by the findings in an earlier study by Ajah *et al*. that compared the prevalence of domestic violence against women in urban versus rural areas in Enugu South-east Nigeria which noted that the proportion of rural women who had experienced GBV was higher than that of the urban women [25]. However, while this study was on persons who volunteered their experience in a facility in a tertiary hospital in Enugu; the participants in the study by Ajah *et al*. were visited in their communities [25]. This could suggest that significant numbers of GBV occurring in the rural areas go unreported, as victims in the rural areas could be less likely to speak up. This is fuelled by the fact GBV remains masked in a culture of silence due to perceived stigmatization from kin and friends and many survivors in their bid to seek justice are blamed, face retribution or ostracized from their families and communities [2, 5, 16, 19].

This study recorded more cases of sexual based gender abuse compared to physical and emotion abuse. Additionally, the proportion of cases of physical/emotional violence (96.29%) to the total cases of GBV per year was higher in 2020 compared to 2019 and 2018. This may be connected with the lockdown in Nigeria occasioned by the ravaging COVID-19 pandemic, which created room for more cases of physical violence. This is in spite of the lower turnout of people to health facilities during the COVID-19 pandemic in 2020 which could probably mean that the number of cases of GBV reported in the year 2020 could have been higher. The finding is also supported by an earlier report that factors such as increased stress, the disruption of social and protective networks, loss of income and decreased access to sexual and reproductive health services occasioned by the COVID-19 pandemic in the 2020 will exacerbate the risk of violence for women [26]. On the other hand, the lower number of cases in 2018 is unclear, but could be due to the introduction of a new recording and reporting forms for use in the unit. It could also be due to increase in awareness of the existence of the GBV unit in the hospital, thereby leading to increase in the number of reported cases with time.

In this study, however, lesser cases of GBV were reported in the year 2020 when compared to the years 2018 and 2019. This reason is unclear, but it may be due to people´s fear of visiting hospitals during the COVID-19 pandemic especially the hospital where the data for this study was collected as it had one of the little COVID-19 isolation and treatment centres in the state. This reduced visits/attendance was also noticed in all other units of the hospital in the year 2020. Surprisingly, the highest number of cases of GBV in the year 2020 was reported in the third quarter (July to September) which coincided with the peak of the first wave of the COVID-19 Pandemic in Nigeria with its attendant restriction of movement while the first quarter (January to March) had the least number of cases.

The study is limited by its sample size, as complete data could only be ascertained from the year 2018 from the unit documenting and handling GBV in the hospital due to the introduction of a recording and reporting tools (register and forms). Furthermore, being a hospital-based study, under estimation of the incidence of GBV is expected as many cases of GBV especially those in the rural areas may not have been recorded in the hospital. There is a paucity of published data on GBV in Enugu, Nigeria.

## Conclusion

GBV is a cause for concern in Enugu Nigeria; affecting mainly young female victims in their prime. There is a trend of increasing number of reported cases of GBV in Enugu Nigeria. Cases of sexual based gender abuse are more prevalent than physical and emotion abuse.

### What is known about this topic

- That Gender based violence (GBV) is a global problem affecting more females than males;
- That the incidence of GBV could be on the increase, but is down-played by issues such as under-reporting of cases by victims.

### What this study adds

- The incidence and pattern of GBV reported in Enugu Nigeria.
